# Surgical treatment of perforative tubercular intestines ulcers at patients with HIV infection

**DOI:** 10.1186/1471-2334-14-S2-P47

**Published:** 2014-05-23

**Authors:** MN Reshetnikov, MS Skopin, RV Mal'tsev, ON Zuban

**Affiliations:** 1 Moscow city scientifically practical center of fight against tuberculosis, Moscow, Russia

## 

High incidence of HIV infection leads to increase in number of tuberculosis cases. The Abdominal Tuberculosis (AT) makes 1/4 among other extra pulmonary localizations at patients with late stages of HIV infection. The worst complication of AT is perforation of tubercular ulcers of intestines when the lethality reaches 80%.

The clinical material is presented by 76 patients with HIV infection in 4B stage and ruptured tubercular intestines ulcers. Depending on localization of ulcers, resection of small intestine or a right-sided hemicolectomy was made.

The first group included 40 patients, the second one - 36. Duration of HIV infection was 7-8 years. The periods from the moment of perforation to operation varied from 12 to 36 hours.

In group 1 primary anastomosis was not made, leaving the created stumps of bringing and taking-away departments of intestines in an abdominal cavity, carrying out a decompression of bringing department of intestines. Relaparotomy was carried out with an interval of 48 hours. After knocking peritonitis two-row anastomosis was made.

In group 2 resection of a gut was finished with one-stage anastomosis, sanitation of an abdominal cavity was made.

Active heavy bilateral processes prevailed among the other forms of lungs tuberculosis. The patients’ number of CD4 was noted to have decreased lower than 150, at this time at the number of CD4 of 55 (72,4%) patients was less than 50.

Tuberculosis colitis at all patients was presented by infiltrative and ulcer form. In 25% of all the cases acid resisting mycobacterium was revealed in the exudate received from an abdominal cavity by luminescent microscopy method. During luminescent microscopy of ulcers edges, mycobacterium tuberculosis was always revealed.

In group 1 the results of the treatment were better: the faster recover from peritonitis was note, and there was no insolvency anastomosis, perforation of new tubercular intestines ulcers was noted at 8 patients. 14 patients delivered in a sad plight with intense immunodeficiency died of accruing polyorgan insufficiency.

In group 2 - 28 patients died, at 18 of which new guts perforation and peritonitis progressing occurred what required repeated surgeries.

